# Error Cancellation During Early Task Performance

**DOI:** 10.1027/1618-3169/a000663

**Published:** 2026-06-22

**Authors:** Sámuel Varga, Joshua Kah Meng Khoo, Denis Cousineau, Jan Derrfuss, Claudia Danielmeier, Roland Pfister

**Affiliations:** ^1^ Experimental Psychology, Department of Psychology, Trier University, Trier, Germany; ^2^ School of Psychology, University of Nottingham, Nottingham, UK; ^3^ École de psychologie, Université d’Ottawa, Ottawa, ON, Canada; ^4^ Institute for Cognitive and Affective Neuroscience (ICAN), Trier University, Trier, Germany

**Keywords:** error cancellation, error processing, performance monitoring, motor inhibition

## Abstract

**Abstract:** Models of performance monitoring hold that following an error, the early postresponse period is devoted primarily to error detection rather than mitigation. However, recent evidence shows that erroneous actions can be terminated within ∼100 ms of their initiation. Prior demonstrations of such error cancellation are based on rapid action slips that emerge in highly over-learned tasks, leaving open the question whether error cancellation generalizes to other types of errors. Here, we probed for the generality of the error cancellation effect by studying medium-speed errors that arise from the incorrect application of a mapping rule in the early stages of implementing a novel instruction. Reanalyses of three publicly available datasets show that error cancellation indeed extends to such errors. Swift error cancellation, therefore, is a remarkably general and automatic component of human performance monitoring.


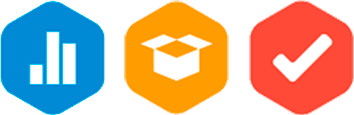


Error monitoring and correction are central to adaptive behavior: By identifying deviations from intended actions, terminating erroneous actions, and adjusting subsequent performance, individuals can prevent similar mistakes in the future. The present study focuses on the termination of erroneous actions, highlighting how the investigation of response termination indicates that error processing is an active and flexible mechanism rather than a purely reactive one.

Traditional approaches to error processing treat errors as discrete events to which the cognitive system reacts. These approaches focus heavily on *reactive* after-effects and adaptive responses, with a strong emphasis on action initiation. Regarding reactive after-effects, research on error detection and posterror adjustments highlights how the cognitive system adapts after an error has already been committed (Danielmeier & Ullsperger, 2011; Dudschig & Jentzsch, 2009; Fiehler et al., 2005; Rabbitt, 1966; Ridderinkhof et al., 2004; Wessel, 2018). In behavioral studies, the most common example of this is posterror slowing (PES), which is the systematic increase in response times for trials immediately following errors (Derrfuss et al., 2021; Pfister & Foerster, 2021; Rabbitt, 1966). The prioritization of action initiation is similarly apparent in modeling work. In ubiquitous drift-diffusion models, for example, evidence accumulates for certain behavioral options, and upon reaching a threshold, a response is emitted (Ratcliff & McKoon, 2008; Wagenmakers et al., 2007; Yang et al., 2020). However, how the emitted response physically evolves and how it is ultimately terminated remain largely unexplored both by behavioral approaches and modeling frameworks.

To complement this picture, recent research has instead explored response termination. In a study by Foerster et al. (2022), participants responded to four centrally located target letters (mapped to two keys) while ignoring distractor letters surrounding the target. The difficulty of the task was further increased by requiring speeded responses. Importantly, the main outcome measure was response duration (RD), defined as the time between keypress and key release (Pfister et al., 2023). Crucially, errors had significantly shorter RDs than correct responses. This effect remained even when controlling for other measures, such as response times (RT) and peak force of the keypress action. Furthermore, shortened RDs for erroneous responses arose mainly after a response reached its peak force. The authors interpreted this result as evidence for the immediate, active termination of an ongoing erroneous motor action. Finally, the finding revealed that this highly efficient cancellation of erroneous actions occurred already within the first 100 ms after action initiation – a timescale previously thought to be reserved for error detection (Foerster et al., 2022).

The rapid timeline for cancellation also coincides with the peaking of the error-related negativity (ERN) in electrophysiological investigations (Falkenstein et al., 1991; Gehring et al., 1993). While the ERN is a highly studied event-related potential, its relation to behavioral measures remains unclear (LoTemplio et al., 2023). Traditionally, the ERN is discussed in terms of signaling error detection (Logan & Crump, 2010; Ridderinkhof et al., 2004) and conflict between correct and erroneous response tendencies (Yeung et al., 2004). Recent results point to an alternative interpretation: Because ERN amplitudes increase as erroneous response durations decrease (i.e., in cases of efficient error cancellation), the ERN may, at least partly, reflect ongoing cancellation efforts (Pfister et al., 2025).

Thus far, the presented evidence for rapid error cancellation is confined to a specific error type. Specifically, in the studies discussed above, errors were associated with substantially shorter RTs than correct responses (Foerster et al., 2022; Pfister et al., 2025). This occurred because previous work addressed speeded responses to simple letter stimuli in a highly over-learned stimulus-response mapping. For these hasty action slips, error cancellation emerged as a robust phenomenon. At the same time, these errors are associated with particularly poor response planning, which may render them especially susceptible to cancellation. Furthermore, these situations include particularly over-learned mapping rules which likely result in strong retrieval and activation of the correct response upon encountering a rule-related stimulus. Depending on current task demands and depending on whether instructions emphasize speed or accuracy (Heitz, 2014; Ratcliff & Rouder, 1998), fast errors may be attributed to impulsivity or response capture (i.e., the involuntary, stimulus-driven activation of an incorrect motor response elicited by the spatial position of the stimulus, Forstmann et al., 2008; Van den Wildenberg et al., 2010). Slow errors, by contrast, emerge when the correct response is not easily detectable or when a task imposes explicit response deadlines (Miletić & Van Maanen, 2019; Van Maanen et al., 2019). A specific instance of the latter can be observed in a setting in which accuracy is rewarded, but failing to respond before a deadline is heavily penalized. Under these conditions, maximizing rewards requires cautious responding, but also gradually reducing the evidence needed to commit to a choice as decision time increases to still meet the response deadline (Murphy et al., 2016). Compared to these types of errors, here we asked whether error cancellation would also occur for medium-speed errors with RTs in a similar range as correct responses. Such medium-speed errors may arise early in task learning, when individuals are working with novel stimulus–response mapping rules and no response deadlines are imposed.

In the current study, we present reanalyses of three publicly available datasets from our laboratory that include early errors in a novel task, while also providing data on response durations (total sample size of *N* = 660 participants). The studies were originally conducted to address a different research question independent of error processing, and the corresponding publication is currently being compiled (Pfister et al., in preparation). In these datasets, participants performed a short two-alternative forced-choice task of 69 trials, and in the current reanalysis, we compared RTs and RDs for correct versus erroneous responses. Error cancellation should emerge as shorter RDs for errors as compared to correct responses, whereas we expected error RTs not to be shorter than those of correct responses. This was indeed the case (see Figure 1).

**Figure 1:**
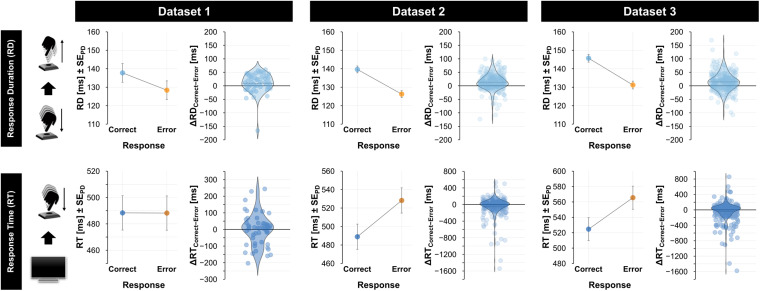
Mean response durations (RDs, upper row) and response times (RTs, lower row) for correct and erroneous responses across three datasets. Error bars show standard errors of paired differences (SE_PD_; Pfister & Janczyk, 2013).

## Dataset 1

The pre-registration for the original study underlying Dataset 1 can be found at: https://aspredicted.org/9k4d-96z7.pdf. The original raw data for all experiments are available at: https://osf.io/hjw5z/. The relevant subset of the data and the analysis script used in the current study are available at: https://osf.io/pwcd2/.

### Method

#### Participants

Previous studies on error cancellation reported large cancellation effects on RDs (*d*_*z*_ = 1.34 in Foerster et al., 2022; and *d*_*z*_ = 1.85 in Pfister et al., 2025). If error cancellation for medium-speed errors occurred with the same effect size as for hastily initiated errors, a sample of N = 10 participants would already provide high power of 1-β > 0.95 (note, however, that the previous effect size estimates rely on a substantially higher number of trials per participant as compared to Dataset 1). The available dataset exceeded this number by including *N* = 60 participants (39 men, 21 women, 0 nonbinary, 54 right-handed, six left-handed, 0 ambidextrous; age: *M* = 39 years, *SD* = 12 years). This sample size allows us to detect an effect size of *d*_*z*_ = 0.37 with 1–β = .80 at *α* = .05.

For all three experiments, participants were recruited via the online crowdsourcing platform Prolific, and the experiment was programmed and administered using E-Prime Go. In accordance with institutional guidelines, formal ethical approval was not required for this study; however, all participants provided informed consent prior to data collection. All experiments were conducted in accordance with the Declaration of Helsinki.

#### Stimuli, Task, and Procedure

Participants completed a number classification task by responding to a digit with a left key if this digit was smaller than 5 and with the right key if the digit was larger than 5. Responses were made by pressing either the F or the J key, with the left or right index finger, respectively. The digit stimulus stayed on screen until the computer registered a keypress response and there was no response deadline.

The experiment included number stimuli from 1 to 9, including the 5 that was not mapped to any response and that was not mentioned in the instructions. The original focus of the experiment was performance in these “rule-free” trials, and the pre-registered analyses will be reported elsewhere (Pfister et al., in preparation). For the present reanalyses, we focused on rule-based trials only, i.e., those trials with any target digit from 1 to 4 or 6 to 9. The entire task comprised 69 trials, 64 of which were rule-based trials and 5 of which were rule-free (target digit 5). The experiment began with a training block of 16 trials (presenting each digit twice, except for the five), followed by an experimental block of 53 trials (five trials with a 5 as stimulus and six trials for each of the remaining digits). Here, we pooled the data across training block and experimental block and removed all rule-free trials from the analyses.

#### Data Selection

We planned to exclude participant data from the analyses if overall accuracy was less than 50%; however, no participant committed that many errors. Seven participants did not commit any errors and thus their data could not be analyzed. Additionally, one participant’s data were excluded from the analysis due to response-coding issues. The primary outcome measures were RT and RD, and we compared each measure between correct and erroneous responses. Before conducting the analyses, trials with technical artifacts where the recorded key release preceded the keypress (i.e., timestamp logging errors resulting in an RD < 0 ms) were filtered out. Filtered RDs represented on average 0.87% of trials per participant (*SD* = 2.50%).

### Results

The mean error rate across participants was 5.16% (*SD* = 4.49%, range = 1.56–30.51%), leaving on average 3.25 error trials per participant for analysis (*SD* = 2.69 trials, range = 1–18 trials).

There was a small but not statistically significant RD difference between correct responses (*M* = 138 ms, *SD* = 29 ms) and errors (*M* = 128 ms, *SD* = 47 ms), *t*(51) = 1.85, *p* = .070, *d*_*z*_ = 0.26, consistent in direction with previous findings (e.g., Foerster et al., 2022, see Figure 1). RTs of correct responses (*M* = 488 ms, *SD* = 94 ms) and errors (*M* = 488 ms, *SD* = 144 ms) were almost identical, *t*(51) = 0.01, *p* = .990, *d*_*z*_ = 0.00.

## Dataset 2

Experiments 2 and 3 were conducted with substantially larger sample sizes (*N* = 300 each) and modified stimulus sets (see below for details). This allowed us to re-evaluate the findings related to Dataset 1. The original study of Dataset 2 was preregistered at: https://aspredicted.org/bhxh-4rdy.pdf.

### Method

#### Participants and Task

A total of *N* = 300 participants took part in Experiment 2 (174 men, 123 women, 3 nonbinary; 268 right-handed, 32 left-handed, 0 ambidextrous; age: *M* = 40 years, *SD* = 12 years), providing 80% power to detect an effect size of *d*_*z*_ = 0.16 at *α* = .05. In contrast to the digit classification task used in Experiment 1, Experiment 2 employed a shape classification task. Participants responded using the left and right arrow keys to indicate whether the presented stimulus was an upward- or downward-pointing triangle. On rule-free trials, a blue circle was presented instead. On average, 0.64% (*SD* = 5.54%) of trials was filtered out because of invalid RDs.

### Results

The data of 13 participants were filtered out for having accuracy rates below 50% (ranging from 21.88% to 48.44%). Additionally, 31 participants did not commit any errors. The mean error rate of the remaining sample was 6.99% (*SD* = 6.84%, range = 1.56–44.44%), with the average number of error trials for analysis being 4.45 (*SD* = 4.34 trials, range = 1–28 trials).

Crucially, the analysis on RDs indicated significant error cancellation, with RDs of erroneous responses (*M* = 126 ms, *SD* = 46) being shorter than those of correct responses (*M* = 140 ms, *SD* = 39), *t*(255) = 7.17, *p* < .001, *d*_*z*_ = 0.45. Additionally, correct responses (*M* = *489* ms, *SD* = 169 ms) were significantly faster than errors (*M* = 528 ms, *SD* = 322 ms), although this difference was numerically small, *t*(255) = −2.88, *p* = .004, *d*_*z*_ = −0.18, with an effect size below common lower bounds for small effects (*d*_*z*_ = 0.20).

## Dataset 3

The preregistration for the original study of Dataset 3 is available at: https://aspredicted.org/fqh3-y4b2.pdf.

### Method

#### Participants and Task

Experiment 3 included 300 participants (175 male, 125 female, 0 nonbinary; 274 right-handed, 26 left-handed 0 ambidextrous; age: *M* = 41 years, *SD* = 13 years), yielding 80% statistical power to detect an effect size of *d*_*z*_ = 0.16 at *α* = .05. The shape classification task was identical to the one of Experiment 2 for rule-based trials, whereas rule-free trials showed a gray hexagon instead of a blue circle. On average, 0.74% (*SD* = 3.35%) of trials were filtered out due to invalid RDs.

### Results

The data of six participants were excluded for having accuracy rates below 50% (range = 27.59–48.44%), and an additional 31 participants did not have any errors to analyze. The mean error rate of the remaining sample was 7.20% (*SD* = 7.07%, range = 1.56–43.75%), with the average number of error trials included in the analyses being 4.57 (*SD* = 4.48 trials, range = 1–28 trials).

The RD comparison again resulted in a significant difference, with erroneous responses (*M* = 131 ms, *SD* = 45) being briefer than correct responses (*M* = 146 ms, *SD* = 36), *t*(262) = 6.87, *p* < .001, *d*_*z*_ = 0.42. As in the case of the previous dataset, the RT comparison between correct responses (*M* = 525 ms, *SD* = 159 ms) and errors (*M* = 566 ms, *SD* = 275 ms) revealed a significant effect, *t*(262) = −2.73, *p* = .007, *d*_*z*_ = −0.17, which, however, did not cross the conventional lower bound for small effects (*d*_*z*_ = 0.20).

### Pooled Analyses

**Figure 2:**
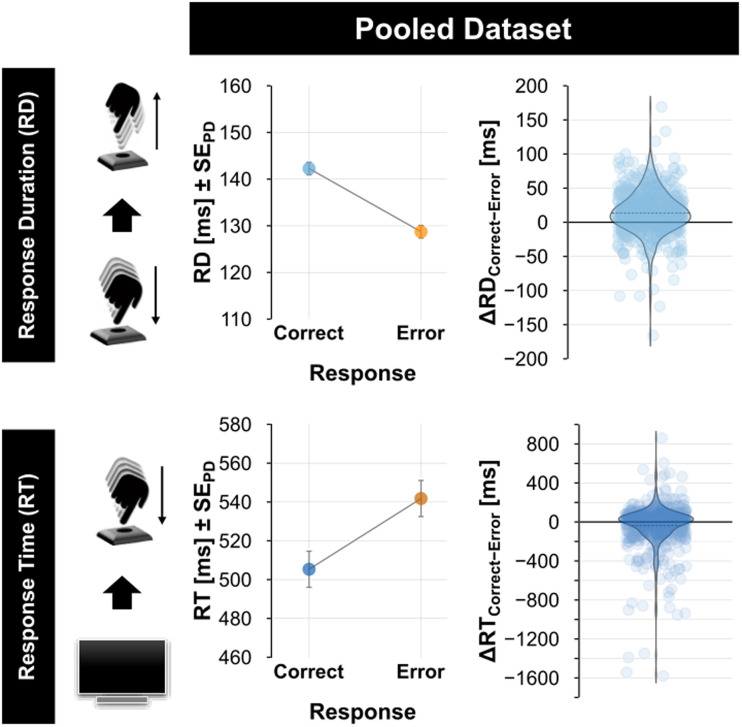
Mean response duration (RD, upper row) and response time (RT, lower row) for correct and erroneous responses in the pooled data of all three datasets. Error bars show standard errors of paired differences (SE_PD_; Pfister & Janczyk, 2013).

To maximize statistical power given the relatively low number of errors in the datasets, we pooled the three datasets and repeated the analyses (see Figure 2). No participant contributed more than one dataset to the pooled analysis.

### Results

The mean error rate of the pooled data was 6.92% (*SD* = 6.79%, range = 1.56–44.44%), leaving on average 4.39 errors per participant for analysis (*SD* = 4.29 trials, 1-28 trials).

**Figure 3:**
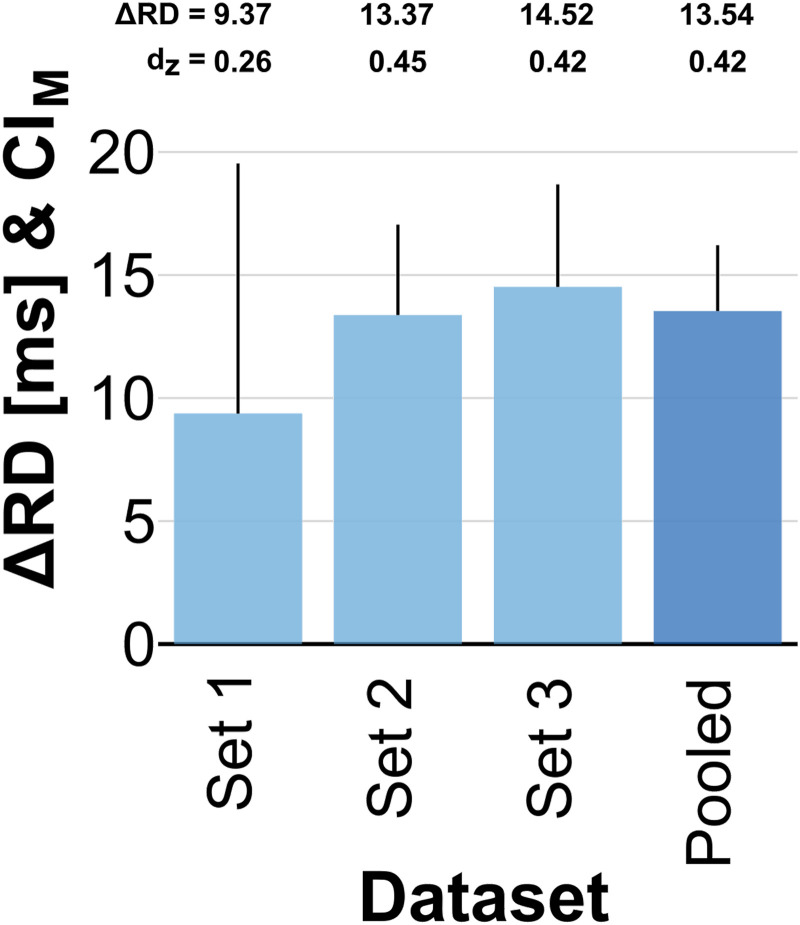
Error cancellation effects in response durations (RDs), computed as RD_correct responses_ minus RD_errors_. Error bars indicate 95% Confidence Intervals (CI_M_) for the mean.

The RD comparison again showed error cancellation: RDs were shorter in errors (*M* = 129 ms, *SD* = 46 ms) than in correct responses (*M* = 142 ms, *SD* = 37 ms), *t*(570) = 9.94, *p* < .001, *d*_*z*_ = 0.42, 95% CI_SM_ = [0.33, 0.50] (see Figure 3). The RT comparison remained statistically significant, with correct responses (*M* = 505 ms, *SD* = 160 ms) being systematically faster than errors (*M* = 542 ms, *SD* = 289 ms), *t*(570) = −3.92, *p* = < .001, *d*_*z*_ = −0.16, albeit with an effect size below the common lower bound for small effects.

## Discussion

In the current study, we sought to investigate whether error cancellation emerges in the case of medium-speed errors or whether it is limited to hasty action slips in the context of strongly over-learned stimulus response mappings as reported in previous studies (Foerster et al., 2022; Pfister et al., 2025). To this end, we reanalyzed three datasets with sufficiently large sample sizes (combined *N* = 660) to analyze errors committed during the early stages of learning a new stimulus–response mapping.

Starting with the RT analyses, although correct and erroneous responses differed significantly (except for Dataset 1), errors were in fact slightly slower than correct responses (albeit not even reaching the conventional threshold for a small effect, i.e., *d*_*z*_ = 0.20, Cohen, 1988). Accordingly, these errors likely reflect the incorrect application of the instructed mapping rather than premature responding. Given the lack of deadlines, they are also plausibly different from *slow errors* attributable to a looming response deadline (Murphy et al., 2016; Van Maanen et al., 2019). Highlighting this distinction motivated our description of the current errors as being *medium-speed*.

Importantly, RD comparisons revealed the cancellation of ongoing erroneous actions also for these medium-speed errors. This pattern indicates that the phenomenon of error cancellation is not limited to hasty action slips. Together, these results are consistent with a two-stage account of error processing that accommodates different error typologies. For the medium-speed errors observed here, Stage 1 is characterized by rule uncertainty during the early stages of task performance, which slightly prolongs error RTs relative to correct RTs. Stage 2 involves the inhibitory and corrective processes that operate during movement execution, which are already active during these early stages and successfully shorten error RDs relative to correct RDs.

This two-stage account can also be applied to the fast action slips observed in previous literature. For fast errors, Stage 1 corresponds to the premature activation of an incorrect motor plan (e.g., via stimulus-driven response capture), leading to exceedingly fast RTs. Yet Stage 2 plausibly remains identical: the same inhibitory and corrective processes detect the mismatch and rapidly truncate the ongoing erroneous movement. In cases where these parallel corrective processes are particularly robust and fast, they may even manifest behaviorally as double responses (Evans et al., 2020; Fiehler et al., 2005), although such responses were too rare in our current paradigm to analyze systematically.

In contrast, this two-stage explanation yields a distinct prediction for deadline-driven slow errors. If a slow error is driven purely by uncertainty and the sudden need to respond before a deadline, the action essentially amounts to a guess. Because there is no latent accumulation of correct information to guide a subsequent corrective process, Stage 2 would be absent. Consequently, one would not expect to observe active error cancellation (i.e., shortened RDs) for true deadline-driven errors. While our findings extend the phenomenon of error cancellation to medium-speed errors, the continuous motor dynamics of slow errors remain unexplored. Future research should explicitly measure response durations in deadline-driven tasks (or perhaps other types of slow errors) to determine whether error cancellation is indeed absent, or whether the phenomenon also applies to slower, nonimpulsive error types.

Our results also highlight the value of RD analyses in general, which extend conventional RT comparisons by revealing diverging patterns, and being especially suitable for studying online performance adjustments. For example, RDs can also be expected to exhibit sensitivity to forthcoming task demands, including planned actions later in an action sequence (Pfister et al., 2023). Finally, it is interesting to note that compared to the large error cancellation effects reported in previous studies (*d*_*z*_ = 1.34 in Foerster et al., 2022; and *d*_*z*_ = 1.85 in Pfister et al., 2025), the effects revealed in the current analyses are markedly smaller (*d*_*z*_ = 0.42, 95% CI_SM_ = [0.33, 0.50]). We suggest that this difference in effect size likely reflects the decreased number of trials available for analysis, thus coming with less stable RT and RD condition estimates for each individual participant (Miller, 2023; Miller & Ulrich, 2016). It is also possible that the nature of the errors themselves, being medium-speed and arising during early stages of task performance, further attenuated the observed effect size.

In conclusion, the current results extend previous findings by demonstrating that error cancellation, as captured by RD, is not only limited to action slips under speeded conditions and highly over-learned stimulus mapping rules but also emerges for slower, mapping-related errors in early stages of task performance. The present observations thus further support the generality of the error cancellation effect, reinforcing the perspective that error processing is an active mechanism rather than a purely reactive one.

## Notes

**Publication Ethics:** In accordance with institutional guidelines, formal ethical approval was not required for this study; however, all participants provided informed consent prior to data collection. All experiments were conducted in accordance with the Declaration of Helsinki.

**Open Science:** Open Data: The original raw data for all experiments are available at: https://osf.io/hjw5z/ (Pfister, 2026).

Open Materials: The relevant subset of the data and the analysis script used in the current study are available at: https://osf.io/pwcd2/ (Varga et al., 2026).

Preregistration: The preregistration for the original study underlying Dataset 1 can be found at: https://aspredicted.org/9k4d-96z7.pdf. The original study of Dataset 2 was pre-registered at: https://aspredicted.org/bhxh-4rdy.pdf. The preregistration for the original study of Dataset 3 is available at: https://aspredicted.org/fqh3-y4b2.pdf.

## References

[c1] Cohen, J. (1988). *Statistical power analysis for the behavioral sciences* (2nd ed.). Lawrence Erlbaum Associates.

[c2] Danielmeier, C., & Ullsperger, M. (2011). Post-error adjustments. *Frontiers in Psychology*, *2*(233), Article 233. 10.3389/fpsyg.2011.00233 21954390 PMC3173829

[c3] Derrfuss, J., Danielmeier, C., Klein, T. A., Fischer, A. G., & Ullsperger, M. (2022). Unbiased post-error slowing in interference tasks: A confound and a simple solution. *Behavior Research Methods*, *54*(3), 1416–1427. 10.3758/s13428-021-01673-8 34713426 PMC9170639

[c4] Dudschig, C., & Jentzsch, I. (2009). Speeding before and slowing after errors: Is it all just strategy? *Brain Research*, *1296*, 56–62. 10.1016/j.brainres.2009.08.009 19679114

[c5] Evans, N. J., Dutilh, G., Wagenmakers, E.-J., & Van Der Maas, H. L. J. (2020). Double responding: A new constraint for models of speeded decision making. *Cognitive Psychology*, *121*, Article 101292. 10.1016/j.cogpsych.2020.101292 32217348

[c6] Falkenstein, M., Hohnsbein, J., Hoormann, J., & Blanke, L. (1991). Effects of crossmodal divided attention on late ERP components. II. Error processing in choice reaction tasks. *Electroencephalography and Clinical Neurophysiology*, *78*(6), 447–455. 10.1016/0013-4694(91)90062-9 1712280

[c7] Fiehler, K., Ullsperger, M., & Von Cramon, D. Y. (2005). Electrophysiological correlates of error correction. *Psychophysiology*, *42*(1), 72–82. 10.1111/j.1469-8986.2005.00265.x 15720582

[c8] Foerster, A., Steinhauser, M., Schwarz, K. A., Kunde, W., & Pfister, R. (2022). Error cancellation. *Royal Society Open Science*, *9*(3), Article 210397. 10.1098/rsos.210397 35296111 PMC8905184

[c9] Forstmann, B. U., Jahfari, S., Scholte, H. S., Wolfensteller, U., van den Wildenberg, W. P. M., & Ridderinkhof, K. R. (2008). Function and structure of the right inferior frontal cortex predict individual differences in response inhibition: A model-based approach. *The Journal of Neuroscience: The Official Journal of the Society for Neuroscience*, *28*(39), 9790–9796. 10.1523/JNEUROSCI.1465-08.2008 18815263 PMC6671204

[c10] Gehring, W. J., Goss, B. M., Coles, M., Meyer, D. E., & Donchin, E. (1993). A neural system for error detection and compensation. *Psychological Science*, *4*(6), 385–390. 10.1111/j.1467-9280.1993.tb00586.x

[c11] Heitz, R. P. (2014). The speed-accuracy tradeoff: History, physiology, methodology, and behavior. *Frontiers in Neuroscience*, *8*, Article 150. 10.3389/fnins.2014.00150 24966810 PMC4052662

[c12] Logan, G. D., & Crump, M. J. C. (2010). Cognitive illusions of authorship reveal hierarchical error detection in skilled typists. *Science*, *330*(6004), 683–686. 10.1126/science.1190483 21030660

[c13] LoTemplio, S., Lopes, C. L., McDonnell, A. S., Scott, E., Payne, B. R., & Strayer, D. L. (2023). Updating the relationship of the NE/ERN to task-related behavior: A brief review and suggestions for future research. *Frontiers in Human Neuroscience*, *17*, Article 1150244. 10.3389/fnhum.2023.1150244 37082151 PMC10110987

[c14] Miletić, S., & Van Maanen, L. (2019). Caution in decision-making under time pressure is mediated by timing ability. *Cognitive Psychology*, *110*, 16–29. 10.1016/j.cogpsych.2019.01.002 30735843

[c15] Miller, J. (2024). How many participants? How many trials? Maximizing the power of reaction time studies. *Behavior Research Methods*, *56*(3), 2398–2421. 10.3758/s13428-023-02155-9 37537492 PMC10991062

[c16] Miller, J., & Ulrich, R. (2016). Optimizing research payoff. *Perspectives on Psychological Science: A Journal of the Association for Psychological Science*, *11*(5), 664–691. 10.1177/1745691616649170 27694463

[c17] Murphy, P. R., Boonstra, E., & Nieuwenhuis, S. (2016). Global gain modulation generates time-dependent urgency during perceptual choice in humans. *Nature Communications*, *7*, Article 13526. 10.1038/ncomms13526 PMC5123079 27882927

[c500] Pfister, R. (2026, January 15). *Guiding rules* [Data]. https://osf.io/hjw5z

[c18] Pfister, R., & Foerster, A. (2022). How to measure post-error slowing: The case of pre-error speeding. *Behavior Research Methods*, *54*(1), 435–443. 10.3758/s13428-021-01631-4 34240334 PMC8863758

[c19] Pfister, R., Foerster, A., Schwarz, K. A., Varga, S., Steinhauser, M., & Kunde, W. (2025). Error‐related brain activity indicates immediate auto‐cancellation of action slips. *Psychophysiology*, *62*(10), Article e70160. 10.1111/psyp.70160 41054872 PMC12501828

[c20] Pfister, R., & Janczyk, M. (2013). Confidence intervals for two sample means: Calculation, interpretation, and a few simple rules. *Advances in Cognitive Psychology*, *9*(2), 74–80. 10.5709/acp-0133-x 23826038 PMC3699740

[c21] Pfister, R., Neszmélyi, B., & Kunde, W. (2023). Response durations: A flexible, no-cost tool for psychological science. *Current Directions in Psychological Science*, *32*(2), 160–166. 10.1177/09637214221141692

[c22] Rabbitt, P. M. (1966). Errors and error correction in choice-response tasks. *Journal of Experimental Psychology*, *71*(2), 264–272. 10.1037/h0022853 5948188

[c23] Ratcliff, R., & McKoon, G. (2008). The diffusion decision model: Theory and data for two-choice decision tasks. *Neural Computation*, *20*(4), 873–922. 10.1162/neco.2008.12-06-420 18085991 PMC2474742

[c24] Ratcliff, R., & Rouder, J. N. (1998). Modeling response times for two-choice decisions. *Psychological Science*, *9*(5), 347–356. 10.1111/1467-9280.00067

[c25] Ridderinkhof, K. R., Ullsperger, M., Crone, E. A., & Nieuwenhuis, S. (2004). The role of the medial frontal cortex in cognitive control. *Science*, *306*(5695), 443–447. 10.1126/science.1100301 15486290

[c26] van den Wildenberg, W. P. M., Wylie, S. A., Forstmann, B. U., Burle, B., Hasbroucq, T., & Ridderinkhof, K. R. (2010). To head or to heed? Beyond the surface of selective action inhibition: A review. *Frontiers in Human Neuroscience*, *4*, Article 222. 10.3389/fnhum.2010.00222 21179583 PMC3004391

[c27] van Maanen, L., Katsimpokis, D., & Van Campen, A. D. (2019). Fast and slow errors: Logistic regression to identify patterns in accuracy–response time relationships. *Behavior Research Methods*, *51*(5), 2378–2389. 10.3758/s13428-018-1110-z 30187434 PMC6797658

[c501] Varga, S., Khoo, J. K. M., Cousineau, D., Derrfuss, J., Danielmeier, C., & Pfister, R. (2026, January 27). *Error cancellation during early task performance* [Materials]. https://osf.io/pwcd2 10.1027/1618-3169/a000663 PMC13334463 42324955

[c28] Wagenmakers, E., Van Der Maas, H. L. J., & Grasman, R. P. P. P. (2007). An EZ-diffusion model for response time and accuracy. *Psychonomic Bulletin & Review*, *14*(1), 3–22. 10.3758/BF03194023 17546727

[c29] Wessel, J. R. (2018). An adaptive orienting theory of error processing. *Psychophysiology*, *55*(3), Article e13041. 10.1111/psyp.13041 29226960

[c30] Yang, C.-T., Cousineau, D., & Pfister, R. (2020). The editorial on the special issue: Are sequential sampling models the future gold standard of cognitive psychology? *The Quantitative Methods for Psychology*, *16*(2), 71–72. 10.20982/tqmp.16.2.p071

[c31] Yeung, N., Botvinick, M., & Cohen, J. D. (2004). The neural basis of error detection: Conflict monitoring and the error-related negativity. *Psychological Review*, *111*(4), 931–959. 10.1037/0033-295x.111.4.939 15482068

